# Dry eye disease and spondyloarthritis: expanding the spectrum of systemic inflammatory disorders associated with ocular surface disease. Data from the international AIDA Network Spondyloarthritis Registry

**DOI:** 10.3389/fmed.2024.1422307

**Published:** 2024-09-17

**Authors:** Antonio Vitale, Valeria Caggiano, Eduardo Martín-Nares, Nunzia Di Meglio, Cristian Sica, Andrea Hinojosa-Azaola, Maria Orsetta Perfetti, Alessandra Pagliara, Giorgia Guidetti, Alex Fonollosa, Roberta Lopez, Jessica Sbalchiero, Jurgen Sota, Ester Carreño, Perla Ayumi Kawakami-Campos, Stefano Gentileschi, Alejandra de-la-Torre, Gian Marco Tosi, Maria Antonietta Mazzei, Alberto Balistreri, Bruno Frediani, Luca Cantarini, Claudia Fabiani

**Affiliations:** ^1^Department of Medical Sciences, Surgery and Neurosciences, Research Center of Systemic Autoinflammatory Diseases and Behçet's Disease Clinic, University of Siena, Siena, Italy; ^2^Azienda Ospedaliero-Universitaria Senese [European Reference Network (ERN) for Rare Immunodeficiency, Autoinflammatory and Autoimmune Diseases (RITA) Center], Siena, Italy; ^3^Department of Immunology and Rheumatology, Instituto Nacional de Ciencias Médicas y Nutrición Salvador Zubirán, Mexico City, Mexico; ^4^Unit of Diagnostic Imaging, Department of Medical, Surgical and Neuro Sciences and of Radiological Sciences, University of Siena, Azienda Ospedaliero-Universitaria Senese, Siena, Italy; ^5^Ophthalmology Unit, Department of Medicine, Surgery and Neurosciences, University of Siena, Siena, Italy; ^6^Department of Ophthalmology, Biocruces Bizkaia Health Research Institute, Cruces University Hospital, University of the Basque Country, Barakaldo, Spain; ^7^Department of Ophthalmology, Hospital Universitario Rey Juan Carlos, Madrid, Spain; ^8^Department of Ophthalmology, Hospital Universitario Fundación Jiménez Díaz, Madrid, Spain; ^9^Department of Ophthalmology, Instituto Nacional de Ciencias Médicas y Nutrición Salvador Zubirán, Mexico City, Mexico; ^10^Neuroscience Research Group (NEUROS), Neurovitae Center for Neuroscience, School of Medicine and Health Sciences, Institute of Translational Medicine (IMT), Universidad del Rosario, Bogotá, Colombia; ^11^Bioengineering and Biomedical Data Science Lab, Department of Medical Biotechnologies, University of Siena, Siena, Italy

**Keywords:** dry eye disease (DED), spondyloarthritis, diagnosis, break-up time (BUT), Schirmer test, ocular surface

## Abstract

**Objective:**

Dry eye disease (DED) is a condition associated with a myriad of systemic disorders. According to recent preliminary data, axial spondylarthritis (axial-SpA) could represent a new entity associated with DED. Therefore, this study aimed to assess DED in patients with axial SpA by performing quantitative and qualitative specific tests to investigate the potential association between DED and ocular surface damage in patients with axial-SpA and to identify potential variables associated with DED.

**Methods:**

A total of 71 patients with axial-SpA who fulfilled the Assessment of SpondyloArthritis International Society (ASAS) classification criteria and 19 healthy controls were enrolled in this study. Both the patients and the controls underwent a complete ocular assessment aimed at evaluating the tear film and ocular surface, which included the Schirmer test, tear break-up time (TBUT), fluorescein staining, and lissamine green staining. The Ocular Surface Disease Index (OSDI) questionnaire was administered to all patients.

**Results:**

DED symptoms were reported in 46 (64.8%) patients and three (15.8%) healthy controls (*p* = 0.0004). The odds ratio for receiving a diagnosis of axial-SpA based on the presence of dry-eye-related symptoms was 9.2 (95% C.I. 2.72–42.52, *p* = 0.001). The Schirmer test values of < 6 mm/5 min were observed in 31 (43.7%) patients with axial-SpA and two (10.5%) healthy controls (*p* = 0.013); a TBUT of <5 s was observed in 34 (47.9%) patients with axial-SpA and six (31.6%) healthy controls. The median OSDI score was found to be 22.9 (IQR = 29.35) among the patients with axial-SpA and 0.0 (IQR = 4.69) among the healthy controls (*p* = 0.009). The fluorescein and lissamine green staining of the ocular surface indicated a significantly higher Oxford Grading Scale in the patients with axial-SpA than in the healthy controls.

**Conclusion:**

Patients with axial-SpA often complain of eye dryness, which may be quantified with the self-administered OSDI questionnaire and objectively assessed through the tests commonly used for the diagnosis of DED. Patients suspected of having axial-SpA should routinely be asked about dry eye symptoms and evaluated for potential corneal and conjunctival damage.

## Introduction

Axial spondylarthritis (axial-SpA) is associated with many inflammatory ocular manifestations, especially episcleritis, uveitis, and scleritis (1–3). In recent times, dry eye disease (DED) has also been associated with axial-SpA, thus widening the list of diseases leading to superficial ocular disorders (4).

Clinical entities capable of inducing dry eye symptoms include oncohematological diseases, infectious conditions (such as hepatitis C virus and human immunodeficiency virus), and autoimmune disorders, such as Sjögren's syndrome, systemic lupus erythematosus, rheumatoid arthritis, immunoglobulin G4-related disease (IgG4-RD), and sarcoidosis. The use of various drugs has been associated with DED, which includes antihistamines, tricyclic antidepressants, selective serotonin reuptake inhibitors, diuretics, beta-blockers, anticholinergic drugs, oral contraceptives, and isotretinoin. Aging is closely linked to DED and so is the female sex (5).

DED is empirically categorized as aqueous-deficient, when symptoms are related to reduced tear production, and as hyperevaporative, when it is caused by increased evaporation of the tear film. However, more than 80% of DED cases show a combination of these two factors (6).

Based on recent evidence of superficial keratopathy and DED in patients with axial-SpA (4), the present study was conducted to assess the presence of symptoms and signs of DED using specific questionnaires, qualitative and quantitative tests of the tear film, and corneal and conjunctival staining aimed at identifying and quantifying the ocular surface damage in such patients.

## Patients and methods

This study with a prospective design enrolled patients with axial-SpA and healthy controls between September 2023 and December 2023. Both the ophthalmologists and the operator conducting the statistical analysis worked blindly.

Patients with axial-SpA who fulfilled the Assessment of SpondyloArthritis International Society (ASAS) criteria (7) were enrolled in the study. Demographic, clinical, and therapeutic data were drawn from the international AutoInflammatory Disease Alliance (AIDA) Network registry dedicated to spondylarthritis (https://aidanetwork.org/en/register/spondyloarthritis). In parallel, a total of 19 healthy controls were enrolled. Patients and controls with the following conditions were excluded from the study: rheumatoid arthritis, Sjögren's syndrome or other autoimmune connective tissue diseases, positive anti-Ro/SSA autoantibodies, positive anti-La/SSB autoantibodies, IgG4-RD, rosacea, HIV infection, HBV infection, HCV infection, syphilis infections, lymphoma and other hematological disorders, and underwent a recent ocular surgery. In addition, patients and controls with smoking habits were also excluded from the study. Specifically, none of the patients who fulfilled the 2016 American College of Rheumatology/European League Against Rheumatism classification criteria for primary Sjögren's syndrome were included in the study (8).

The participants enrolled did not apply topical eyedrops at the time of the ocular examination and 1 month before the examination. Both patients and controls underwent a complete ocular assessment, which included the evaluation of the tear film and the ocular surface. Data were collected from the right eye of each enrolled participant.

The primary aim of the study was to further evaluate whether patients with axial-SpA experience dry eye symptoms. The secondary aim of the study was to investigate the potential association between DED and ocular surface damage in patients with axial-SpA and to identify any variables associated with DED in such cases.

The level of tear secretion was assessed using the Schirmer test and the tear break-up time (TBUT) test. A Schirmer strip (I-DEW tearstrips; Tottenham Lane, London, UK) was placed over the lower eyelid margin, positioned midway between the middle and outer thirds, for a duration of 5 min without the use of topical anesthesia. The participants were instructed to keep their eyes closed during the procedure. The Schirmer test was subsequently conducted under conditions suitable for evaluation (9). The TBUT was assessed following the application of a preservative-free solution (Contacare Ophthalmic & Diagnostics, Vadodara District, Gujarat, India) containing 1% fluorescein dye (administered in a volume of 1 ml) into the conjunctival sac using a micropipette. The participants were then instructed to blink several times. The TBUT was determined by taking three consecutive measurements using a stopwatch, and the average of these three values was computed (10). DED was defined as abnormal tear production, as determined by the Schirmer test (< 6 mm and < 10 mm after 5 min), or as abnormal tear film stability, as determined by the TBUT (< 5 and < 10 s) (11, 12).

Ocular surface damage was assessed using fluorescein and lissamine green staining. In both cases, the ocular surface was divided into three zones, which comprised the nasal conjunctiva, cornea, and temporal conjunctiva. The fluorescein and lissamine green staining scores ranged from 0 to 5 for each zone according to the Oxford Grading Scale, with 0 representing the lower intensity of the staining and 5 representing the most severe (13, 14).

The Ocular Surface Disease Index (OSDI) questionnaire, a 12-item validated questionnaire designed to provide a rapid assessment of ocular surface symptoms, was administered to all participants to assess the ocular symptoms of dryness (15).

The clinical variables included in the analysis were sex; age at the time of ocular assessment; age at the start of ocular symptoms; age at the onset of articular manifestations (in axial-SpA patients); age at the time of diagnosis of axial-SpA; age at the start of the treatment for axial-SpA; axial-SpA disease duration; axial-SpA treatment duration; presence of psoriasis, anterior uveitis, inflammatory bowel diseases, autoimmune thyroiditis, human leukocyte antigen (HLA)-B27 positivity; use of selective serotonin reuptake inhibitors (SSRI) or serotonin-norepinephrine reuptake inhibitors (SNRI); treatment with topical glucocorticoids, systemic glucocorticoids, and conventional disease-modifying anti-rheumatic drugs (cDMARDs); and use of biotechnological agents.

All patients and healthy controls provided signed informed consent for the study, which was conducted according to the Declaration of Helsinki. The study was approved by the Ethics Committee of the Azienda Ospedaliero-Universitaria Senese, Siena, Italy in June 2019 (Ref. N. 14951).

Descriptive statistics were used to present the sample size, percentage, frequency count, mean, median, standard deviation, and interquartile range (IQR). Pairwise comparisons between the patients with axial-SpA and healthy controls were conducted using Student's *t*-test or the Mann–Whitney U test for quantitative data, and the χ^2^ test or Fisher's exact test for qualitative data, as appropriate. A univariate binomial logistic regression analysis was performed on axial-SpA patients to identify any associations between the Schirmer test in the right eye < 6 mm as the dependent variable and other demographic, clinical, and therapeutic variables assessed in this study. β1 estimates from the univariate binomial logistic regression, corresponding to the log of odds ratios, were also provided. A multiple linear regression analysis was conducted on both patients with axial-SpA and healthy controls to adjust for the association between the Schirmer test values in the right eye (dependent variable) and the diagnosis of axial-SpA (independent variable) for the age at ocular symptom onset and the use of SSRI/SNRI (independent covariates). All these statistical analyses were two-tailed and were performed using RStudio software version 4.3.0, with a type I error set at 0.05 (*p* < 0.05).

The odds ratios for the “Schirmer test in the right eye < 6 mm” between the patients with axial-SpA and healthy controls, along with corresponding 95% confidence intervals (95% C.I.), were calculated using Episheet software (16).

## Results

In total, 71 patients with axial-Spa and 19 healthy controls were included. Clinical features of the patients with axial-SpA are summarized in Table 1.

**Table 1:** Demographic data, clinical details, and therapeutic history of patients with axial spondylarthritis enrolled in this study.

**Demographic data and clinical features**	
Sex, female (%)	57 (80.3)
Age at time of ocular examination (years), mean ± SD	45.54 ± 15.38
Ocular symptoms, n (%)	46 (64.8)
Age at ocular symptom onset (years), mean ± SD	35.11 ± 16.09
Disease duration (years), median (IQR)	6 (13.8)
Treatment duration at the ocular tests (years), median (IQR)	1 (2)
Psoriasis, *n* (%)	19 (26.8)
Anterior uveitis^#^, *n* (%)	10 (14.1)
Undifferentiated IBD, *n* (%)	7 (9.9)
Crohn's disease, *n* (%)	1 (1.4)
Ulcerative rectocolitis, *n* (%)	1 (1.4)
HLA-B27^*^, *n* (%)	6/53 (11.3)
**Treatments**	
SSRI/SSNI use, *n* (%)	10 (14.1)
Glucocorticoids use, *n* (%)	9 (12.7)
cDMARDs use, *n* (%)	13 (18.3)
• Methotrexate, *n* (%)	10 (14.1)
• Sulfasalazine, *n* (%)	3 (4.2)
Median treatment duration with cDMARDs, years	1 (2)
Anti-TNF agents, *n* (%)	33 (46.5)
• Adalimumab, *n* (%)	25 (35.2)
• Etanercept, *n* (%)	3 (4.2)
• Certolizumab Pegol, *n* (%)	2 (2.8)
• Golimumab, *n* (%)	2 (2.8)
• Infliximab, *n* (%)	1 (1.4)
Median treatment duration with anti-TNF agents (IQR), years	1 (2)
Topic treatment, *n* (%)	19 (26.8)
**Most frequent comorbidities (frequency** > **1)**	
Hashimoto's thyroiditis, *n* (%)	6 (8.5)
Endometriosis, *n* (%)	3 (4.2)
Fibromyalgia, *n* (%)	2 (2.8)
Adenomyosis, *n* (%)	2 (2.8)
Hypertension, *n* (%)	2 (2.8)

cDMARDs, conventional disease-modifying anti-rheumatic drugs; HLA, human leukocyte haplotype; IBD, inflammatory bowel diseases; IQR, interquartile range; SD, standard deviation; SNRI, serotonin-norepinephrine reuptake inhibitor; SSRI, selective serotonin reuptake inhibitors; TNF, tumor necrosis factor. ^*^Investigated in 53 patients; ^#^not present at the time of the Schirmer test and the tear break-up time test.

DED symptoms were reported in 46 (64.8%) patients and 3 (15.8%) healthy controls (*p* = 0.0004). The odds ratio for receiving a diagnosis of axial-SpA based on the presence of dry eye-related symptoms was 9.2 (95% C.I. 2.72-42.52, *p* = 0.001).

**Figure 1:**
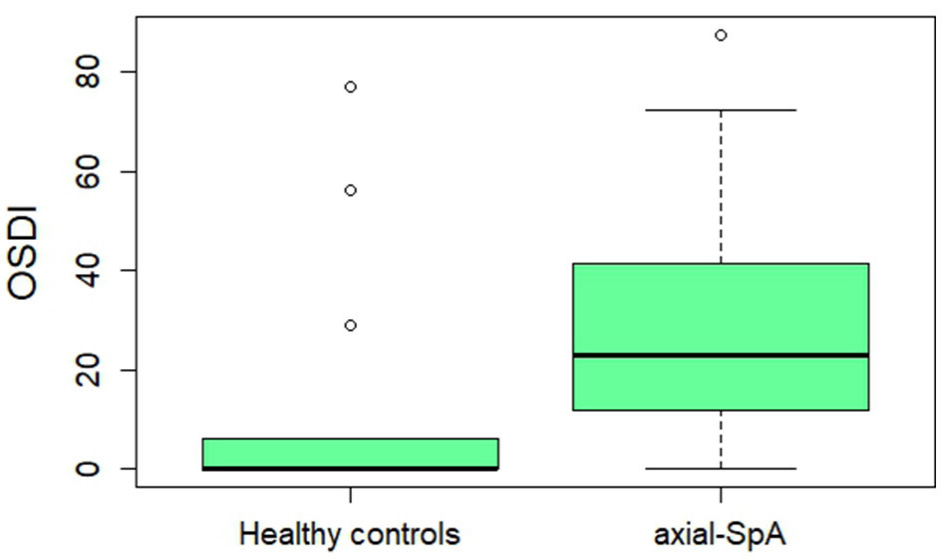
Histograms providing information about the distribution of the Schirmer test values observed in the right eye in 5 min among patients with axial spondylarthritis **(A)** and healthy controls **(B)**, along with the tear film break-up time values among axial spondyloarthrits (axial-SpA) patients **(C)** and the healthy controls **(D)**. axial-SpA, axial spondyloarthritis; TBUT, tear break-up time.

**Figure 2:**
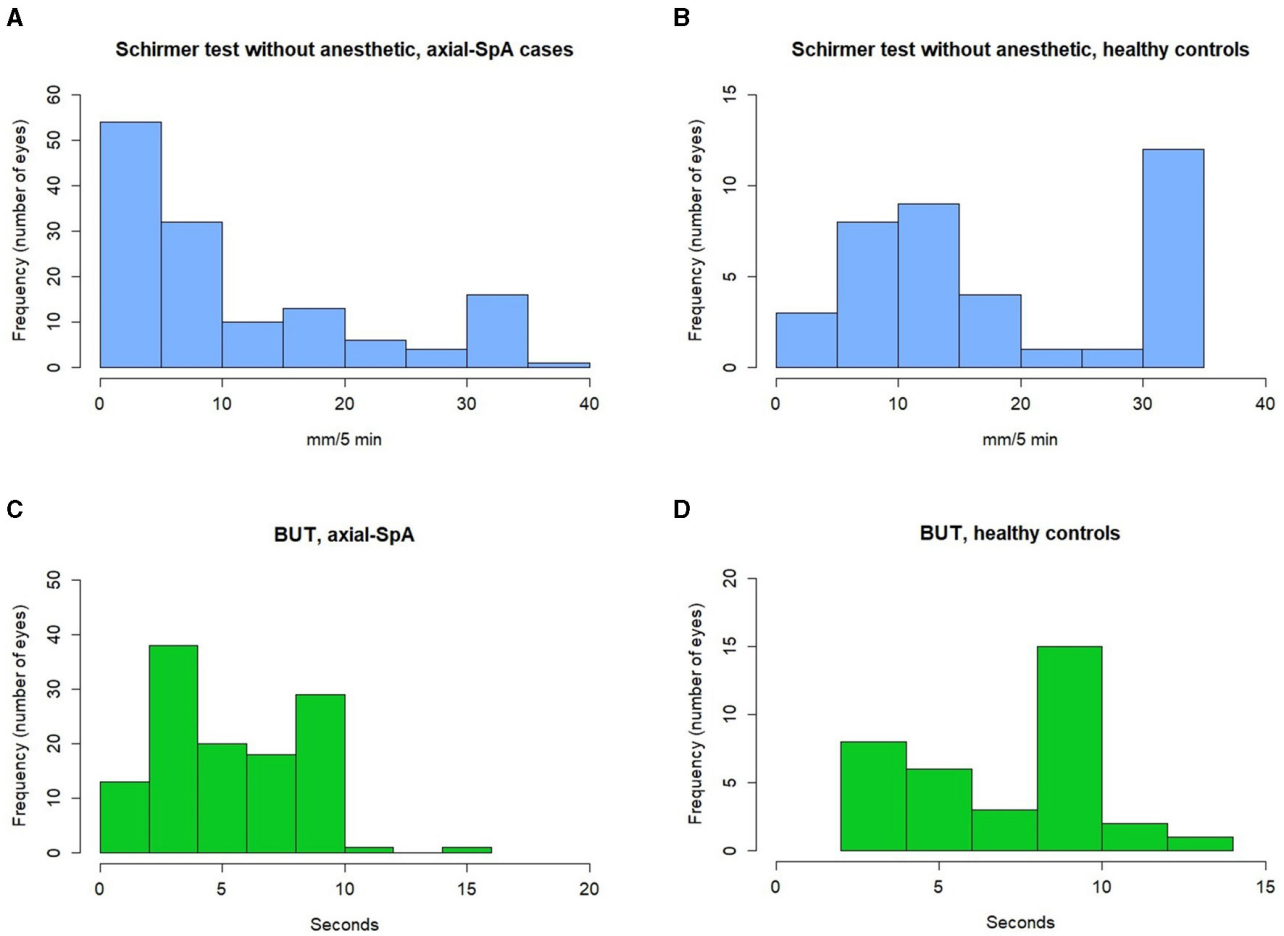
A boxplot describing the Ocular Surface Disease Index (OSDI) in healthy controls and patients with axial-SpA. Thick horizontal lines indicate the median values in the two study groups, while the whiskers indicate 1.5 times the interquartile range; isolated dots indicate the outliers.

The median Schirmer test value calculated in both eyes was 7.00 mm (IQR = 16.25) among patients with axial-SpA and 15.00 mm (IQR = 25) among healthy controls (*p* < 0.0001). The median TBUT value in the total number of eyes from patients with axial-SpA was 5 s (IQR = 6), while the median TBUT value in the total number of eyes from healthy controls was 9 s (IQR = 5) (*p* = 0.006). Figure 1 provides information about the distribution of the Schirmer test and TBUT test values among patients with axial-SpA and healthy controls. Table 2 shows the frequency of axial-SpA patients and healthy controls with the Schirmer test values < 10 mm/5 min and < 6 mm/5 min, as well as with the TBUT test < 10 mm and < 5 mm in the right eye. The median OSDI score was found to be 22.9 (IQR = 29.35) among patients with axial-SpA and 0.0 (IQR = 4.69) among healthy controls (*p* = 0.009). Figure 2 represents the graphical presentation of the OSDI score in the two study groups.

**Table 2:** Pathological Schirmer test and tear break-up time.

	**Axial-SpA**	**Healthy controls**	***p*-value**
Schirmer test < 10 mm/5 min	40 (56.3%)	5 (26.3%)	0.028
Schirmer test < 6 mm/5 min	31 (43.7%)	2 (10.5%)	0.013
TBUT < 10 s	8 (42.1%)	51 (71.8)	0.011
TBUT < 5 s	6 (31.6%)	34 (47.9%)	0.009

Frequency of the Schirmer test values < 10 mm/5 min and < 6 mm/5 min and of TBUT < 10 s and < 5 s among patients with axial spondyloarthritis and healthy controls. Axial-SpA, axial spondylarthritis; TBUT, tear break-up time.

**Figure 3:**
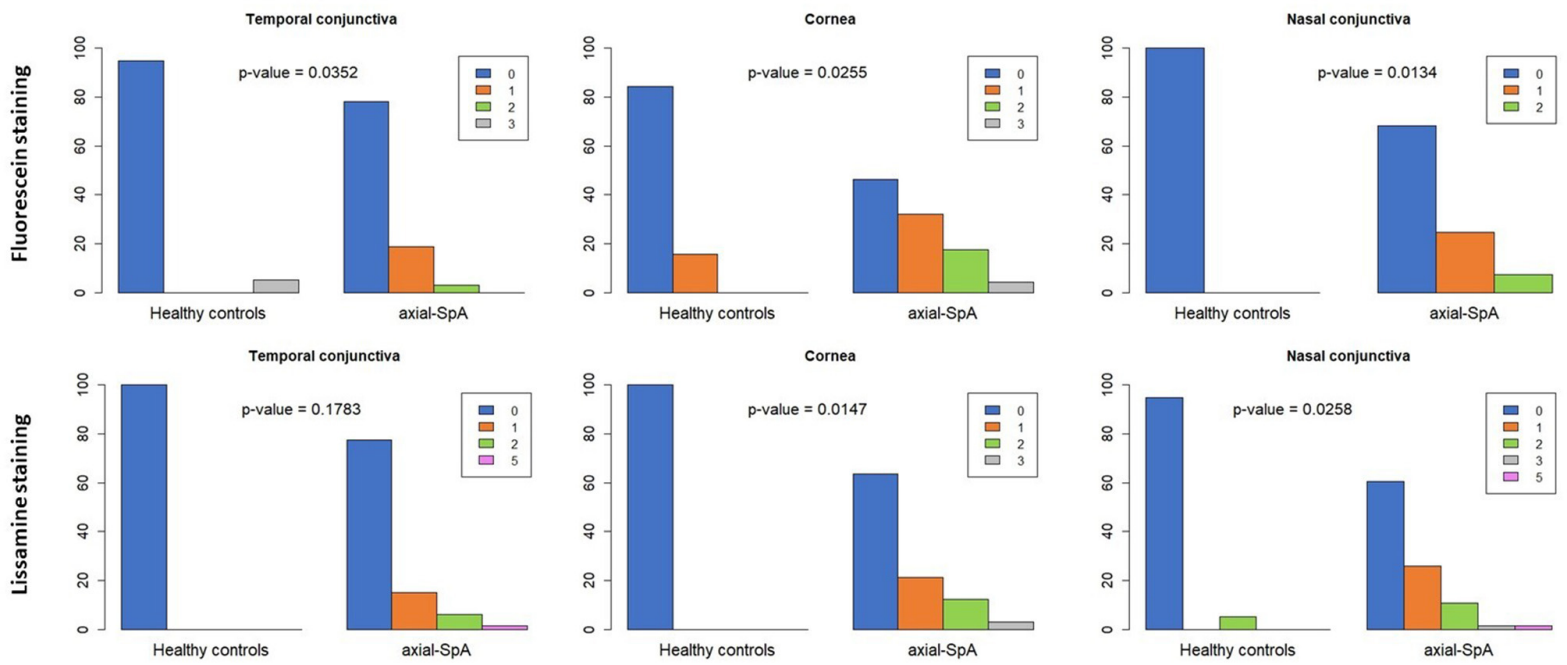
The Oxford Grading Scale with fluorescein staining and with lissamine green staining in the right eyes of healthy controls and patients with axial-SpA. The Oxford score ranged from 0 to 5 for each zone (temporal conjunctiva, cornea, and nasal conjunctiva), with 0 representing the absence of damage and 5 representing the most severe damage.

**Figure 4:**
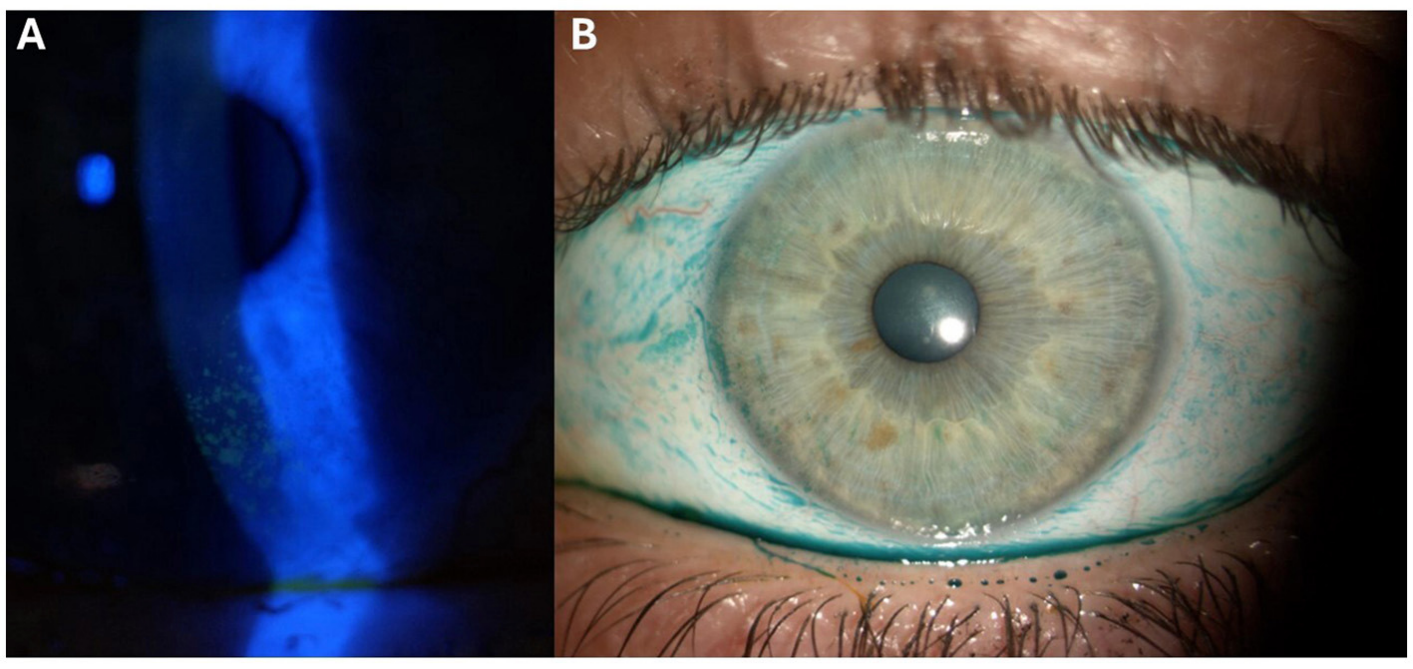
Fluorescein and lissamine green staining patterns on the ocular surface of a patient included in the study: **(A)** Representative image displaying the fluorescein staining of the corneal surface, showcasing patchy patterns primarily localized in the inferior hemi-cornea, consistent with a Grade 3 severity based on the Oxford Grading Scale (13, 14); **(B)** image depicting the lissamine green staining on the ocular surface, encompassing the nasal and temporal conjunctiva, as well as the cornea, demonstrating damage indicative of a Grade 3 severity based on the Oxford Grading Scale (13, 14).

Figure 3 provides information about the Oxford Grading Scale with fluorescein staining and lissamine green staining in the right eyes of the patients with axial-SpA and healthy controls. Figure 4 shows a case of ocular surface damage in a patient with axial-SpA.

Supplementary Table 1 provides information about the univariate binomial regression analysis conducted between the Schirmer test < 6 mm in the right eye (dependent variable) and demographic, clinical, and therapeutic data of patients with axial-SpA (independent variables). The use of SSRI/SNRI and other drugs leading to DED was significantly associated with the identification of the Schirmer test < 6 mm (*p* = 0.027), while the age at ocular symptom onset showed a trend toward significance (*p* = 0.057).

Supplementary Table 2 provides information about the linear regression analysis investigating any association between the Schirmer test values in the right eye (dependent variable) and the demographic, clinical, and therapeutic data of patients with axial-SpA (independent variables). The age at the onset of dry eye symptoms was inversely associated with the Schirmer value in the right eye (*p* = 0.023).

The crude odds ratio for the Schirmer test < 6 mm between the patients with axial–SpA and healthy controls was 6.59 (95% C.I. 1.41–30.68, *p* = 0.008). The odds ratio was 5.16 (95% C.I. 1.27–34.93, *p* = 0.04) after adjusting for age at the time of ocular assessment, 6.32 (95% C.I. 1.60–42.35, *p* = 0.02) after adjusting for sex, 6.53 (95% C.I. 1.94–64.56, *p* = 0.01) after adjusting for the use of SSRI/SSNI and other drugs leading to DED, 7.73 (95% C.I. 1.99–51.34, *p* = 0.009) after adjusting for the concomitant presence of anterior uveitis (in the right eye), 6.43 (95% C.I. 1.63–43.05, *p* = 0.02) after adjusting for the presence of psoriasis, 6.47 (95% C.I. 1.66–43.06, *p* = 0.02) after adjusting for the presence of inflammatory bowel diseases, and 6.14 (95% C.I. 1.57–40.86 *p* = 0.02) after adjusting for the presence of Hashimoto's thyroiditis.

## Discussion

The present study further supported the presence of DED and ocular surface damage in patients with axial-SpA and demonstrated that it may be observed during both the Schirmer test and the TBUT test. Moreover, the Oxford Grading Scale with fluorescein staining and lissamine green staining confirmed previously reported data about ocular surface damage in axial-SpA (4). The median OSDI score highlighted the impact of DED symptoms in patients with axial-SpA compared to healthy controls.

Since DED was identified both through the Schirmer test and the TBUT, our results suggest that this condition may be observed using both exams, indicating a mixed nature, hyposecretive and hyperevaporative, of DED in patients with axial-SpA. The Schirmer test measures the secretions of the lacrimal gland, while the TBUT describes the stability of the tear film and is reduced in hyperevaporative DED (6).

The degree of DED severity was both evident and notable; indeed, the Schirmer test resulted not only in < 10 mm/5 min with a significantly higher frequency among the patients with axial-SpA than among controls but also resulted in < 6 mm/5 min. Similarly, the patients not only exhibited a TBUT of < 10 with a significantly greater frequency than controls but also a TBUT of < 5. Accordingly, the Oxford Grading Scale demonstrated a substantial degree of damage to the ocular surface, which corroborated the data proposed by Lee et al. about the significantly higher frequency of superficial keratopathy in the ankylosing spondylitis population compared to the control group (4).

Dry eye symptoms assessed with the OSDI score proved to be significantly more impactful on the quality of life among the patients with axial-SpA than among controls (17). In addition, the presence of dry eye-related symptoms was significantly more frequent among the patients with axial-SpA, with the risk of being diagnosed with axial-SpA being more than eight times higher (Odds Ratio 9.2) among patients reporting dry eye symptoms compared to controls. This finding suggests that it would be advisable to include questions about the presence of any dry eye-related symptoms in cases of suspected axial-SpA during the patient interview, in addition to the well-established questions regarding inflammatory back pain and the presence of items included in the ASAS classification criteria for axial-SpA (7).

The results of this study included axial-SpA in the list of rheumatologic diseases to be investigated in patients with DED. In total, 64.8% of axial-SpA patients reported symptoms associated with DED, with 56.3% demonstrating a positive Schirmer test value. These percentages are similar or even higher than those observed in other immune-related rheumatologic diseases, including rheumatoid arthritis (38–47%), systemic lupus erythematosus (13.4–39.5%), systemic sclerosis (37–79% of patients), sarcoidosis (19.2%), and IgG4-related disease (22%) (18–20).

The univariate regression analysis was performed to identify factors associated with the Schirmer test values and the Schirmer test value < 6 mm/5 min among axial-SpA patients to identify variables that were predictive of more pronounced DED in these patients. The age at ocular symptom onset showed a potential effect on both the Schirmer test values and the Schirmer test value < 6 mm/5 min; similarly, as expected, the use of SSRI/SNRI and other drugs leading to DED were significantly associated with a Schirmer test value of < 6 mm. Therefore, as patients' age and psychotropic drugs are known to be associated with DED (5), the association of DED with spondylarthritis was adjusted for the age of the participants included in the study at the time of ocular assessment and for the use of concomitant drugs potentially inducing DED. In addition, considering the sex differences associated with DED, an adjustment for sex was also carried out (21). None of these adjustments resulted in a loss of statistical significance in the risk of observing the Schirmer test < 6 mm/5 min between axial-SpA patients and healthy controls, confirming that the diagnosis of axial-SpA was associated with DED even after adjusting for age of the tested participants, the use of DED-inducing drugs, and sex. Therefore, a close collaboration between ophthalmologists and rheumatologists should be established when dealing with patients with DED, even when classical immune-mediated disorders commonly associated with DED, such as Sjögren's syndrome and rheumatoid arthritis, have been ruled out.

In the present study, no specific association was observed between the Schirmer test values or the frequency of the Schirmer test values < 6 mm/5 min and the duration of articular disease in axial-SpA patients. This finding could signify that DED manifestations may be independent of the duration of articular disease, but it could also suggest that ocular issues may begin before the onset of the joint component. Neither the use of anti-tumor necrosis factor biotechnological agents nor the use of cDMARDs seemed to have an impact on DED in terms of the Schirmer test; however, this may be due to a reduced duration of therapy, with a median treatment duration of only 1 year. However, the investigation of the effectiveness of treatments used for spondyloarthritis on comorbidities in axial-SpA patients represents a current and intriguing issue that could also encompass DED, warranting the inclusion of a greater number of patients for long-term follow-up (22).

A clinical relationship between DED and uveitis exists as both ocular conditions can appear concomitantly in patients of any age and with any uveitis etiology (23). Anterior uveitis, commonly identified in patients with axial-SpA (24), was found in approximately 14% of our patients with axial-SpA but it did not influence DED, which appeared to be independent of the concurrent presence or absence of uveitis.

Of note, cutaneous psoriasis, which may be associated with axial-SpA in the recognized subtype of psoriatic axial-SpA (24), has also been linked to DED (25–27). Similarly, inflammatory bowel diseases, which are also well-known to be associated with axial-SpA, have been identified as an independent risk factor for DED and ocular surface damage (28). The results from the regression analysis and statistical adjustment analysis conducted in this study suggest that DED may be observed regardless of the history of cutaneous psoriasis. This finding supports previous studies that did not identify a clear association between psoriasis and DED in terms of the pathological Schirmer test and TBUT (29, 30). Similarly, we observed DED in axial-SpA regardless of the coexistence of inflammatory bowel diseases.

The main limitation of the study lies in the small sample size. In addition, this study solely focused on assessing DED in axial-SpA, without including patients with only peripheral seronegative arthritis and patients with psoriatic arthritis. However, this prospective study clearly included axial-SpA in the list of disorders capable of being associated with DED and highlighted the need for further investigations on this issue. Translational studies are needed to understand the cause of DED in these patients to gain a better understanding of how this comorbidity can be treated. It would have been interesting to identify any association or correlation between the severity of DED and the activity of the axial-SpA disease. However, correlating the Schirmer test or the TBUT with a specific data point obtained at the time of the ophthalmological assessment, such as the Ankylosing Spondylitis Disease Activity Score (ASDAS) or the Bath Ankylosing Spondylitis Disease Activity Index (BASDAI) (31, 32), did not seem adequate for this purpose. Conversely, it would have been useful to correlate ophthalmologic variables with magnetic resonance imaging scores related to radiographic progression. However, these data are not available in our cohort at the moment. Future studies should also aim to evaluate potential differences in ocular surface involvement between axial-SpA, peripheral seronegative arthritis, and psoriatic arthritis in the development of DED. Psoriatic arthritis is a chronic disease associated with various extra-articular manifestations and multiple comorbidities. To decide whether to include DED among these comorbidities, it is be necessary to further strengthen the collaboration between rheumatologists and ophthalmologists to enhance the multidisciplinary approach required for the optimal management of this condition (33).

In conclusion, axial-SpA patients often complain of dry eye symptoms, which may be quantified with the self-administered OSDI questionnaire and objectively assessed through the tests commonly used for the diagnosis of DED. Patients suspected of having axial-SpA should routinely be asked about dry eye symptoms and evaluated for potential corneal and conjunctival damage.

## Data Availability

The raw data supporting the conclusions of this article will be made available by the authors, without undue reservation.
