# Supplementary material for: Dry eye disease and spondyloarthritis: expanding the spectrum of systemic inflammatory disorders associated with ocular surface disease. Data from the international AIDA Network Spondyloarthritis Registry
Source: Front Med (Lausanne). 2024 Sep 17;11:1422307. doi: 10.3389/fmed.2024.1422307 (PMC11442350; doi:10.3389/fmed.2024.1422307)
Supplement: Supplementary file 1 [file Table_1.DOCX]

**Supplementary table 1**. Results from univariate binomial regression analysis associating the occurrence of a Schirmer test <6 mm in the right eye with demographic, clinical and therapeutic features from axial spondylarthritis patients. *Abbreviations: Axial-SpA, axial spondylarthritis; cDMARDs, conventional disease modifying anti-rheumatic drugs; HLA, human leukocyte haplotype; SNRI: serotonin-norepinephrine reuptake inhibitor; SSRI, selective serotonin reuptake inhibitors; TNF, tumor necrosis factor.*

| Independent variables | *p*-value (β1 estimate) |
| --- | --- |
| Sex | 0.862 (-0.1054) |
| Age at the ocular examination | 0.096 (0.02780) |
| Age at articular symptoms onset | 0.172 (0.02141) |
| Age at ocular symptoms onset | 0.057 (0.03561) |
| Age at axial-SpA diagnosis | 0.077 (0.03115) |
| Age at the start of treatment of axial-SpA | 0.234 (0.01974) |
| Axial-SpA disease duration | 0.704 (0.008858) |
| Treatment duration | 0.417 (0.02833) |
| Presence of psoriasis | 0.720 (-0.2025) |
| Presence of anterior uveitis | 0.520 (-0.4855) |
| Presence of intestinal involvement | 0.243 (-1.00017) |
| Presence of HLA-B27 | 0.719 (0.3137) |
| Presence of Autoimmune thyroiditis | 0.156 (1.2417) |
| SSRI/SSNI use | **0.027 (1.8563)** |
| Glucocorticoids use | 0.172 (1.0296) |
| cDMARDs use | 0.513 (0.4210) |
| Anti-TNF use | 0.532 (-0.3302) |
| Previous topical ocular treatment | 0.186 (0.7239) |

**Supplementary table 2.** Results from linear regression analysis investigating any association between the Schirmer test values in the right eye and the demographic, clinical and therapeutic features of patients with axial spondylarthritis. *Abbreviations: Axial-SpA, axial spondylarthritis; cDMARDs, conventional disease modifying anti-rheumatic drugs; HLA, human leukocyte haplotype; SNRI: serotonin-norepinephrine reuptake inhibitor; SSRI: selective serotonin reuptake inhibitors; TNF, tumor necrosis factor.*

| Independent variables | *p*-value (β1 estimate) |
| --- | --- |
| Sex | 0.584 (-2.006) |
| Age at the ocular examination | 0.138 (-0.14459) |
| Age at articular symptoms onset | 0.181 (0.12286) |
| Age at ocular symptoms onset | **0.023 (-0.22494)** |
| Age at axial-SpA diagnosis | 0.148 (-0.1478) |
| Age at the start of treatment of axial-SpA | 0.219 (-0.11926) |
| Axial-SpA disease duration | 0.940 (-0.01055) |
| Treatment duration | 0.799 (-0.04927) |
| Presence of psoriasis | 0.432 (2.612) |
| Presence of anterior uveitis | 0.993 (-0.04433) |
| Presence of intestinal involvement | 0.385 (4.116) |
| Presence of HLA-B27 | 0.587 (-2.780) |
| Presence of Autoimmune thyroiditis | 0.306 (-4.824) |
| SSRI/SSNI use | 0.16 (-5.580) |
| Glucocorticoids use | 0.361 (-3.872) |
| cDMARDs use | 0.19 (-4.774) |
| Anti-TNF use | 0.388 (2.501) |
| Previous topical ocular treatment | 0.0938 (-5.418) |
